# Supplementary material for: Finite element analysis of a one-piece zirconia implant in anterior single tooth implant applications
Source: PLoS One. 2020 Feb 24;15(2):e0229360. doi: 10.1371/journal.pone.0229360 (PMC7039452; doi:10.1371/journal.pone.0229360)
Supplement: Attachment — Submitted filename: Responses.docx [file pone.0229360.s002.docx]

Responses to Journal Editor and Reviewers:

Responses to Journal Editor

1. When submitting your revision, we need you to address these additional requirements.

Please ensure that your manuscript meets PLOS ONE's style requirements, including those for file naming. The PLOS ONE style templates can be found at

[http://www.journals.plos.org/plosone/s/file?id=wjVg/PLOSOne_formatting_sample_main_body.pdf](http://www.journals.plos.org/plosone/s/file?id=wjVg/PLOSOne_formatting_sample_main_body.pdf) and [http://www.journals.plos.org/plosone/s/file?id=ba62/PLOSOne_formatting_sample_title_authors_affiliations.pdf](http://www.journals.plos.org/plosone/s/file?id=ba62/PLOSOne_formatting_sample_title_authors_affiliations.pdf)

Response: The title page and manuscript main text have been revised accordingly.

2. In your methods section you write that the study used CBCT scans from an implant clinic database with patient identifiers removed. Please provide the name of the database and clarify whether the data were anonymized or de-identified before you accessed them.

Response: The CBCT database is our in-house Virginia Commonwealth University School of Dentistry CBCT database. We asked our staff to send us one dataset anonymized prior to utilizing it in our study. A clarification statement was added.

“The first step of the workflow (Fig. 1) to create the *in silico* three dimensional FEA models was selecting CBCT scans from Virginia Commonwealth University (VCU) School of Dentistry internal radiology database with patient identifiers removed prior to the utilization of the scans.”

- In your data availability statement you write, "All relevant data are within the paper." Please ensure you have provided the individual data points used to create the figures and determine means, medians and variance measures presented in the results, tables and figures and provided details of the database from which the underlying images were obtained such that other researchers can replicate the analyses ([http://journals.plos.org/plosone/s/data-availability#loc-faqs-for-data-policy](http://journals.plos.org/plosone/s/data-availability#loc-faqs-for-data-policy)).

Response: We added the raw measurement and statistical data from the FEA models into the supplementary data.

3. Please amend either the abstract on the online submission form (via Edit Submission) or the abstract in the manuscript so that they are identical.

Response: Done.

4.  We note that Figure(s) [#] in your submission contain copyrighted images. All PLOS content is published under the Creative Commons Attribution License (CC BY 4.0), which means that the manuscript, images, and Supporting Information files will be freely available online, and any third party is permitted to access, download, copy, distribute, and use these materials in any way, even commercially, with proper attribution. For more information, see our copyright guidelines: [http://journals.plos.org/plosone/s/licenses-and-copyright](http://journals.plos.org/plosone/s/licenses-and-copyright).

We require you to either (1) present written permission from the copyright holder to publish these figures specifically under the CC BY 4.0 license, or (2) remove the figures from your submission:

1.         You may seek permission from the original copyright holder of Figure(s) [#] to publish the content specifically under the CC BY 4.0 license.

We recommend that you contact the original copyright holder with the Content Permission Form ([http://journals.plos.org/plosone/s/file?id=7c09/content-permission-form.pdf](http://journals.plos.org/plosone/s/file?id=7c09/content-permission-form.pdf)) and the following text:

“I request permission for the open-access journal PLOS ONE to publish XXX under the Creative Commons Attribution License (CCAL) CC BY 4.0 ([http://creativecommons.org/licenses/by/4.0/](http://creativecommons.org/licenses/by/4.0/)). Please be aware that this license allows unrestricted use and distribution, even commercially, by third parties. Please reply and provide explicit written permission to publish XXX under a CC BY license and complete the attached form.”

Please upload the completed Content Permission Form or other proof of granted permissions as an "Other" file with your submission.

In the figure caption of the copyrighted figure, please include the following text: “Reprinted from [ref] under a CC BY license, with permission from [name of publisher], original copyright [original copyright year].”

Response: Fig 1 has been modified to remove copyrighted images. We only preserved the software’s names in the workflow.

2.    If you are unable to obtain permission from the original copyright holder to publish these figures under the CC BY 4.0 license or if the copyright holder’s requirements are incompatible with the CC BY 4.0 license, please either i) remove the figure or ii) supply a replacement figure that complies with the CC BY 4.0 license. Please check copyright information on all replacement figures and update the figure caption with source information. If applicable, please specify in the figure caption text when a figure is similar but not identical to the original image and is therefore for illustrative purposes only.

Response: See above.

Reviewer #1: This article numerically investigates one-piece zirconia implant in anterior single tooth implant applications. The paper presented in a poor quality. The results are NOT clearly explained. The structure of the paper does NOT follow any specific method. From reviewer's point of view, the article is required more than a major revision. In the following, there are comments that are appropriate to reinforce the article.

Response: We appreciate this insight. The manuscript has been revised significantly including the data collection, results, discussion and conclusion.

- The paper needs keywords.

Response: We are sorry that the keywords are no appearing in the manuscript. The following keywords are placed in the PLoS ONE submission system:

**Keyword:** Dental Implants, Finite Element Analysis, Fracture, Loading, Zirconia

- The subtitles should be numbered.

Response: Changes have been made.

- The quality of the figures should be increased.

Response: The figures have been formatted with resolution and size based on PLoS ONE guideline.

- The graphs should be presented in a standard way.

Response: New graphs were made in the standard format.

- The reviewer is unable to follow the paper goal.

Response: The last paragraph of the introduction has been revised. Additional statements were added. The paragraph now read:

“This study applied FEA to examine the effects of different clinical scenarios that had not been studied biomechanically. This includes a healed socket (HS), reduced bone width edentulous site (RB), and extraction socket with bone grafting (EG) on the peri-implant osseous structure around a one-piece Zir implant. This study utilizes high resolution segmentation from CBCT to create its simulated osseous structures for the various scenarios studied. The rationale of the study was that the different mechanical properties of Ti and Zir may influence the distribution of von Mises stress and equivalent strain on heterogenous peri-implant osseous structures. The null hypothesis then is that Zir implants loaded with different forces in certain situations would transmit those forces into the surrounding peri-implant bone with similar magnitude and distribution as the Ti implants. By accepting or rejecting the null hypothesis that there is no difference between Ti and Zir on peri-implant bone and graft, the goal of the study is to aid the clinician in establishing a rationale for use of Zir implants in various clinical scenarios a patient may present with in the anterior maxilla.”

- The figures need captures.

Response: Captures have been added to the figures.

Reviewer #2: The scientific paper propose a detailed FE-based numerical analysis of one-piece zirconia implant concerning three clinically simulated models.
The authors present a very interesting study. This manuscript is well structured using a rich introduction concerning state of the art with respect to scientific literature overview.
The abstract is correct synthesised in order to understand the study goals and the principal obtained results. I would like to recommend this paper for publication in the journal “PLOS ONE“. The major drawbacks are itemized below:

Line 81: It is suggested to replace "The Authors" by "Marcián et al.".

Response: We appreciate the suggestion and have made the changes per the request.

Line 149: "direcrtion" ?

Response: We appreciate the suggestion and have made the changes per the request.

The type of the strain (true strain, equivalent strain, elastic strain, etc. ?) used in the entire manuscript should be specified.

Response: Clarifications were made throughout the paper -> stress measured as von Mises Stress (MPa), strain measured as equivalent strain; in some aspects the “stress/strain” nomenclature was maintained as it was previously explained what that nomenclature meant.

Quality of the figures 3a-3b in pdf version of a manuscript is very poor. The values in legends are illegible.

Response: New figures were made.

Conclusions are not adequately supported by the data presented. It is suggested to add more conclusions based on the results found.

Response: New conclusion statements were made (see below).

“Based on the three FEA models, healed edentulous site (HS), vertical periodontal defect under compression (RB), and immediate extraction with bone grafting site (EG), the following conclusion can be made. Due to the stiffness of the material and its intrinsically high elastic modulus, Zir implants transmit less von Mises stress and induce lower equivalent strain to the peri-implant bone compared to Ti implants. This was statistically significant when cervical cortical bone (HS and RB models) and graft (EG model) were evaluated for one-piece Zir implant compared to its Ti counterpart. Therefore, the peri-implant bone surrounding Zir implants may be less prone to mechanically induced biologic resorption.”

Reviewer #3: This is an interesting study, in which force transmission to bone was investigated in loaded Zir and Ti implants using finite element analysis models. Authors showed that loaded Zir implant transmit less force to bone compared to similar Ti implant, which is presumably to the differences in the stiffness between these two materials.
The experimental procedure is described in sufficient details. However, the statistical analysis is missing in the paper. It is not clear, how many experiments were made and how the differences in the transmission force were assessed.
Data on the force transmission should be presented as mean+/-SD (S.E.M).
In the results section, there are several sentences like “Strain on the implant surface for Ti-6Al-4V averaged at 4.00x10-4 and 1.78x10-4, respectively. These values were higher 69.39% and 62.32 respectively, than Zir which averaged at 1.94x10-4 and 9.33x10-5…”. Such constructions are little confusing, because it is not clear what is taken as 100 %. In this case, values for Ti are about two times higher than those for Zir, and therefore the difference could be estimated as about 100 %. This text, as well as similar passages in other places, should be rephrased. It is not necessary to mention the differences in %, because it can be estimated by readers themselves.

Response: Additional measurements and data sampling were made. Most importantly, standard deviation as well as calculated p-value from n = 10 sampled points within each region assessed (ie. cortical, trabecular, graft, implant) were added

Minor comments
The quality of graphs is low, text is difficult to read. The resolution of figures should be improved.
I would recommend writing Ti6Al4V with the numbers in lowercase; the abbreviation Ti-6Al-4V is confusing, because the numbers seem to be related to wrong atoms.

Response: The reviewer is correct. Changes were made accordingly.
