# Supplementary material for: Finite element analysis of a one-piece zirconia implant in anterior single tooth implant applications
Source: PLoS One. 2020 Feb 24;15(2):e0229360. doi: 10.1371/journal.pone.0229360 (PMC7039452; doi:10.1371/journal.pone.0229360)
Supplement: Attachment — Submitted filename: R2_RESPONSES TO REVIEWERS_PLOS1_01232020.docx [file pone.0229360.s003.docx]

RESPONSES TO REVIEWERS

Reviewer #1: The paper still needs a major revision. It should be mentioned that the author should addressed and replied to the reviewer comments carefully, because the comments are just to improve the paper. In the following, there are comments that are appropriate to reinforce the article.

RESPONSE: We appreciate the time and thorough review.

**Abstract**
- Abstract is too long.

RESPONSE: Abstract was shortened.

- Some of the most important research results should be mentioned at the end of this section.

RESPONSE: We agreed. The end of the abstract is now read.

“Zir implants respond to occlusal loading differently then Ti implants. Zir implants may be more favorable in non-grafted edentulous or immediate extraction with grafting.”

- The authors mentioned “This FEA modeling was completed on the basis to aid in conducting and validating future clinical studies”. The reviewer think this is not a result.

RESPONSE: The sentence was removed.

**Keywords**
- Each paper needs keywords. The keywords are the main technical words that used in paper. For example, you can use following keywords: Finite element analysis; One-piece Zirconia Implant; titanium alloy; Zir implant.

RESPONSE: We agreed. The following keywords were added.

“Finite Element Analysis; One-piece Zirconia Implant; Titanium Alloy; Zircoina Implant.”

**Conclusion**
- This section should be rewritten. It is suggested to use at least 11 line to explain the conclusion. Reader usually check the abstract and conclusion.

RESPONSE: Additional information was added in the conclusion to complement the Abstract. The conclusion is now read:

“In general Zir implants behaves more favorable then Ti implants in terms of peri-implant stress distributions. Three different FEA models, healed edentulous site (HS), vertical periodontal defect under compression (RB), and immediate extraction with bone grafting site (EG), mimicking the common clinical scenarios suggested the following conclusion. Due to the stiffness of the material and its intrinsically high elastic modulus, Zir implants transmit less von Mises stress and induce lower equivalent strain to the peri-implant bone compared to Ti implants. This was statistically significant when cervical cortical bone (HS and RB models) and graft (EG model) were evaluated for one-piece Zir implant compared to its Ti counterpart. Therefore, the peri-implant bone surrounding Zir implants may be less prone to mechanically induced biologic peri-implant bone resorption. Zir implants may be considered for not only for its esthetic properties, but also the stress modulation properties of the material.”

**General comments**
- The subtitles should be numbered.

RESPONSE: There was no subtitle in this manuscript.

- The quality of the figures should be increased.

RESPONSE: We improved the figure resolution as suggested.

- The legend of figure 3 is not clear. Most of the paper use figures with wide of 3.5 inch, or 7 inch. It is recommended to check the quality of figures in this size in your paper.

RESPONSE: We improved the figure resolution and size as suggested.

- The graphs should be presented in a standard way, meaning that the format of the legend, horizontal and vertical axis should be clear with high quality. If you are working with EXEL, please go to the dimension part and use 3.5inx3.5in for your graph. A standard file is attached for instance.

RESPONSE: We improved the graph format resolution and added the legends as suggested.

- The figures need captures.

RESPONSE: Captures were added into the Figures per request.

- In vertical axis of the graphs, unite for stress is MPa, and it is not MPA.

RESPONSE: Changes have been made.
